# Surgery

**Published:** 1876-04

**Authors:** 


### II. Surgery.

1. *The Operation for Hydrocele under Antiseptic Treatment.* Prof. Volkmann. (*Berl. Klin. Wochenschr.*, 1876, No. 3.)

No operation, Prof. V. thinks, is better adapted to convince any skeptic observer of the great usefulness of the antiseptic treatment than the operation for hydrocele. This apparently trifling operation was usually followed by an uncommonly violent reaction and the patient did not recover till after several weeks of profuse suppuration. Now, under the antiseptic dressing, the testicle heals in per primam between the edges of the gaping pouch of the tunica vaginalis ; a small narrow strip of its surface is exposed, which soon is covered by granulations and concealed in the cicatrix. Not the slightest trace of any local reaction can be observed, and on the fifth or sixth day after the operation the patient can leave the sick-bed and walk about, to be further on treated as a dispensary patient. V. has thus treated seventeen cases without having had any ill success. In not a single case had he observed phlegmon or an inflammatory œdema; in six cases there was no fever at all; in three cases an increase of the temperature was noticed only once, *i.e.*, on the evening following the operation; in four cases very moderate fever (temperature not exceeding 39° C.), and in two cases only the thermometer showed a temperature of 40° and 40.5° C.

The complete obliteration of the cavity, *i. e.*, a complete agglutination of the two surfaces of the tunica vaginalis, was noticed at the first change of the dressing in sixteen cases; no secretion could be squeezed out when the dressing was removed. In one case only the agglutination did not take place all over, a small pouch was left, into which a drainage tube was inserted, but after a few days the secretion ceased, the tube could be removed, and the patient recovered as fast as the others.

During the operation, as well as when changing the first bandages, all the precautions required by the truly antiseptic method were strictly observed. The genitals were shaved and thoroughly washed with a watery solution of carbolic acid. Under the spray the sac was opened by a large incision; the cavity was rinsed out with a watery solution of carbolic acid (3 per cent.), and under a continued spray the tunica vaginalis was carefully and accurately stitched on to the skin. For these sutures —and 15 or 20 of them were usually applied—Volkmann used the finest silk; bleeding arteries, no matter how small, were ligated with catgut. The subsequent dressing had to accomplish two purposes, to wit, it should, by a strong and continuous pressure, bring about a smooth and accurate adaptation of the tunica vaginalis to the testicle, while at the same time, it caused the wound to gap, and it should be antiseptic. The cool spray constantly applied while the stitches were put in, and the repeated washing with a cold lotion of carbolic acid in the most cases, excited a strong contraction of the scrotum and caused the desired diminution of the cavity.

The wound then having been washed once more, the scrotum was wrapped up in a number of loops of carbolized gauze, each loop about two to three inches wide; these strips being covered by a large piece of gauze the whole dressing was secured in its place by roller-bandages of carbolized gauze. To this end, the whole lower abdominal region must be bandaged up, and in order to make the dressing really safe, great care must be taken in filling the folds between scrotum and thigh with antiseptic cotton, held in by the carbolized gauze roller. The first dressing remained undisturbed two, three, four days, and sometimes even longer. After two or three changes, the adhesion of the tunica vaginalis was so firm that Lister's antiseptic dressing could be abandoned for a single layer of salicylic cotton, wrapped around the scrotum and held there by a simple suspensory bag. With this dressing the patient could leave the bed and walk about.

2. *New Sound and Extractor for Urinary Calculus.* WAITZ. (*Berlin Klin. Wochenschr.; Centralb. f. Chir.*, 1876, No. 4.)

A London dentist suffered from all the signs of a calculus in the bladder, yet the physicians were unable, by sounding, to discover the stone. He then constructed for himself a sound to find the stone, and an instrument to remove it, and both appliances proved a perfect success to him. The sound was coated with well polished lead, and then blackened by an immersion into a solution of nitrate of silver so that the slightest touch of a hard body would leave a mark on it. By this instrument the presence of a very small calculus was proved. For its removal he employed a fine rubber tube with a funnel-shaped mouth at one end. A silver catheter open at both ends was slipped over that tube in such a way that the rolled-up funnel was near the internal end of the catheter and this end closed by a plug of cacao butter which would melt as soon as it arrives inside of the bladder. The catheter introduced, the rubber tube was pushed on, until its funnel could fully open itself in the cavity of the bladder. The funnel expanded, the urine began to pass through the rubber tube, and the current thereby created in the bladder carried the small calculus into the funnel-mouth of the rubber tube. When the urine ceased to flow and the rubber tube was withdrawn, its funnel closed itself over the calculus carrying it along, and at the same time, protecting the mucous membrane of the urethra against being wounded by the stone.

Such a tube might be very useful for removing the sharp fragments of stones after lithotrity.

3. *Abdominal Sections for Intussusception.* (*The Lanc N.Y. Med. Record*, Feb. 26, 1876.)

At a recent meeting of the Royal Medical and Chirurgical Society, three papers were read upon this operation.

Mr. Howard Marsh related a successful operation, performed by himself on an infant seven months old. The child had been complaining for thirteen days.

The bowel was found projecting two inches beyond the anus, and the ileo-cæcal valves could be seen at the extremity of the protrusion, while in the abdomen a firm cylindrical tumor was felt. Insufflation and distension of the large intestines with lukewarm water, failed to reduce the intussusception. Chloroform having been administered, the abdomen was opened to the extent of two inches in the median line, just below the umbilicus. The intussusception was reduced by first withdrawing the bowel from the abdominal cavity. At least one-half of the colon and an equal part of the small intestine were invaginated. The wound was closed with hare-lip pins and superficial sutures. No bad symptoms followed.

Mr. Marsh was of the opinion that in this case the intestine was merely invaginated for thirteen days, and that inflammation set in twelve or fourteen hours before the operation. All other means failing, he urged the necessity of undertaking the operation, (1) in acute cases of not more than twelve or fourteen hours duration ; (2) in chronic cases in which there had been no symptom of inflammation or strangulation of the intestines.

The second case was under the care of Dr. Hilton Fagge and Mr. H. G. Howse. This case, an adult with intussusception, without symptoms of strangulation, had inflation performed three times unsuccessfully. Mr. Howse opened the abdomen through the umbilicus. The length of the invaginated bowel was eighteen inches. The patient recovered without a bad symptom. In this case, hæmorrhage from the bowels was absent, and in their remarks the two gentlemen showed that it was of great importance not to delay the operation till hæmorrhage occurred.

Mr. Hutchinson related a third and fatal case where the operation was performed upon an infant six months old. The intussusception involved the whole length of the colon and the ileo-cæcal valve. Owing to difficulties encountered in replacing the intestines, punctures were made in two or three places, with a hare-lip needle. Death occurred from peritonitis six hours later. Mr. Hutchinson attributed the fatal issue to the punctures.

4. *Abdominal Section for Intussusception.* (*The Doctor*, Feb. 1, 1876.)

The paper, after reviewing at some length, the discussion at the Medico-Chirurgical Society, quoted above, mentions some cases of obstruction of the bowels from various causes that have been recently related in various periodicals. This operation for intussusception in properly selected cases will be as fruitful in its results as ovariotomy has been. The triumphs of the latter, however, having paved the way for the operation in question, the latter will not, in its onward march to a more settled place amongst operations in surgery, meet with so many obstacles as did its pioneer —ovariotomy.

Mr. Royes Bell, at King's College Hospital, performed in October, abdominal section on a boy sixteen months old for intussusception following diarrhœa. It was impossible to liberate the intestine by traction, either at the time or on post-mortem examination, for the case ended fatally, although an artificial anus was made at the wound. There were but few adhesions.

Mr. Warren Tay has treated cases by injections of water or air while the child is held up by the heels, as advised by Mr. Hutchinson.

Dr. B. F. Seabury records a case of recovery in the *Boston Med. and Surg. Jour.*, of Dec. 2nd. It occurred to a man aged 49 years, and lasted eighteen days, when sloughing took place and a portion of the ileum fourteen inches long was discharged, after which the patient made a rapid recovery.

Dr. Eberhart relates a similar case in *Philadelphia Med. and Surg. Rep.*, of Jan. 8. In this case a portion of small intestine, eighteen inches long, was found. The patient recovered, and took carriage exercise for nearly three months, when constipation, followed by peritonitis, set in and proved fatal. The omentum was found to have adhered in such a manner as to form part of the channel of the bowels.

M. Ortille, of Lille, reports in the *Abeille Médicale* his second case of intussusception cured by swallowing shot mixed with oil.

Two cases are reported in the *Canadian Journal of Med. Science*, in which Dr. Ross tried inversion, combined with the use of quicksilver. In the first of these, other means having failed, the child, aged 18 months, was inverted by an assistant, and five ounces of metallic mercury injected into the rectum, the assistant being desired to shake the patient up and down for about ten minutes, then to incline her buttocks to the right, gradually bringing the body to the horizontal position upon the right side, then turning her upon her face, keeping up the shaking motion. This had probably occupied twenty minutes, when the child's countenance manifested relief. In the erect position the mercury escaped into a basin. Permanent recovery ensued.

In the case of another child, aged 18 months, Dr. Ross adopted the same treatment as in the previous case and with equally good results. The treatment occupied about half an hour.

5. *Anæsthetization During Sleep.* (*The Canada Medical Record*, Feb., 1876.)

Dr. Cluness reports in the *Pacific Med. Journal* two cases of successful chloroformization during sleep.

The first case was that of a little girl, aged eight years, in whom, as a sequel to acute otitis media, the mastoid cells of one side became inflamed. Chloroform was administered upon a four by six piece of surgeon's lint, held as near the child's mouth as possible during sleep without coming in actual contact. Not the slightest effort was made by the child to avoid the inhalation of the anæsthetic, and in a few moments she was well under its influence.

The second case was on the person of a little girl 2½ years old, for the purpose of removal of supernumerary toes.

6. *Fracture of Neck of Femur and both Trochanters.* Welmart. (*Louisville Med. News*, Jan. 29 ; *La Presse Méd., Belge*, No. 14.)

This case is of peculiar interest, owing to complete recovery, although it occurred in a patient aged seventy-two. The fracture healed in ten weeks, without the use of any bandage, with slight shortening and some impairment of rotation and mobility. The patient died about six months after from marasmus, when the diagnosis and result were confirmed by dissection.

7. *Operation for Relief of Phymosis.* Anger. (*La France Méd.*, No. 11.)

The outline of the prepuce, designed to be left upon the penis, is traced with ink. An assistant with two pairs of forceps, one above and one below, lightly holds the prepuce without too much traction, which would make the skin yield more than the mucous membrane. A double-waxed thread is then passed tightly around the prepuce over the line traced by the ink. With this thread serving as a conductor, the skin and mucous membrane are cut with a pair of curved scissors at a single stroke, in front of the ligature.

The resulting advantages are said to be: absence of hæmorrhage, exact and rapid (two days) union of the edges of the skin and mucous membrane, and the doing away with the necessity for etherization and special instruments.

8. *Two Cases of Salivary Calculi.* Pirondi. (*La France Méd.*, No. 11.)

A man, 36 years old, had an abscess open spontaneously in the mouth to the left of the inferior maxilla. A purulent saliva thereafter continued to flow, with swelling and interference with speech and deglutition. Broquier found a sublingual calculus, enlarged the wound and removed a stone as large as an almond. The orifice of Wharton's duct was intact, and communicated with the *bed* of the calculus.

A naval captain had a large salivary stone in the left Whartonian duct. The mucous membrane was completely glued to the calculus, while the orifice of the canal was intact, though slightly dilated. Pirondi largely incised the membrane (respecting the orifice) in the direction of the antero-posterior diameter (longest) of the stone. Using an inflexible spatula as a lever, he then enuncleated the stone which was oblong and weighed more than 8 grammes. Its largest diameters were 4 and 2½ centimetres. In its lowest portion there was a somewhat deep canaliculus which, extending for 4 millimetres, nearly covered the gland.

Both cases were promptly and perfectly cured without any subsequent interference with the function of this part of the salivary apparatus.

9. *Extraction of a Hair-pin from the Female Bladder through the Vagina.* PANAS. (*La France Méd.*, No. 17.)

A girl, 19 years old, introduced a hair-pin into her urethra, so deeply that it could not be withdrawn. Cystitis, hypogastric pain, frequent desire to urinate and burning sensations of the part ensued. After the patient entered hospital, an unsuccessful attempt was made to remove the foreign body by the lithotrite. The bladder was empty, and, the patient not being under the influence of anæsthetics, great pain was endured.

The next day, chloroform was administered, the bladder injected with tepid water, and the lithotrite again introduced. The hair-pin was readily detected upon the floor of the bladder and near the orifice of the urethra, in a vertical position. The jaws of the instrument were directed toward one extremity of the pin, in order to change its position and bring the rounded part in front. It was slightly displaced but not to such a degree as to admit of its extraction.

Panas then withdrew the lithrotite and inserted a pair of dressing forceps, seizing the pin at its presenting part. At the same time with left index finger in the vagina, he felt the points of the pin through the the vesico-vaginal partition, directed backward. While pressing with the finger upon these points, in order to facilitate the extraction, one leg of the pin pierced the vesico-vaginal septum and entered the vagina. The operator seized the leg with a pair of dressing forceps, separated it widely from its companion, using the septum as a fulcrum of leverage, and, converting the hair-pin into a piece of straight wire, easily extracted it from the vagina.

The cure was complete, no lesion of the septum orother part remaining.

Denucé once extracted a crochet needle from the female bladder in the same manner, no fistula resulting. Panas suggests that, in case of necessity, *both* legs might be forced through the septum, the pin straightened, and then removed, the two points of puncture being no more dangerous than one.

